# Auxiliary variables in multiple imputation in regression with missing X: a warning against including too many in small sample research

**DOI:** 10.1186/1471-2288-12-184

**Published:** 2012-12-05

**Authors:** Jochen Hardt, Max Herke, Rainer Leonhart

**Affiliations:** 1 Medical Psychology and Medical Sociology, Clinic for Psychosomatic Medicine and Psychotherapy, University of Mainz, Duesbergweg 6, Mainz 55128, Germany; 2 Social Psychology and Methods, University of Freiburg, Engelberger Straße 41, Freiburg, 79106, Germany

**Keywords:** Multiple imputation, Auxiliary variables, Simulation study, Small and medium size samples

## Abstract

**Background:**

Multiple imputation is becoming increasingly popular. Theoretical considerations as well as simulation studies have shown that the inclusion of auxiliary variables is generally of benefit.

**Methods:**

A simulation study of a linear regression with a response Y and two predictors X_1_ and *X*_2_ was performed on data with n = 50, 100 and 200 using complete cases or multiple imputation with 0, 10, 20, 40 and 80 auxiliary variables. Mechanisms of missingness were either 100% MCAR or 50% MAR + 50% MCAR. Auxiliary variables had low (r=.10) vs. moderate correlations (r=.50) with X’s and Y.

**Results:**

The inclusion of auxiliary variables can improve a multiple imputation model. However, inclusion of too many variables leads to downward bias of regression coefficients and decreases precision. When the correlations are low, inclusion of auxiliary variables is not useful.

**Conclusion:**

More research on auxiliary variables in multiple imputation should be performed. A preliminary rule of thumb could be that the ratio of variables to cases with complete data should not go below 1 : 3.

## Background

Missing data in statistical analyses is the rule rather than the exception. Many statistical methods can only analyse cases with complete data, so a way to deal with the missing data needs to be found. The traditional approach was to only use cases complete data for the variables of interest is fully available, which is often referred to as the Complete Cases analysis (CC). However, not only is CC inefficient when models with many variables are to be examined, it can also introduce bias to regression coefficients when the mechanism that leads to missing data is anything other than missing completely at random [MCAR: [1]. Multiple imputation (MI) was introduced in the 1970s as a way to deal more efficiently with missing data [2]. It met with small to moderate resonance at first, but since 2008, Mackinnon [3] has observed a drastic increase in articles on applying multiple imputation to data analyses published in four leading medical journals (BMJ, JAMA, Lancet, NEJM). However, CC is still utilized in the majority of publications – Karahalious *et al.*[4] reviewed the methods utilized in prospective cohort studies with samples larger than n=1000 published during the first ten years of the century: of 82, only 5 utilized MI.

Basically, MI creates multiple datasets that are copies of the original complete data. The missing observations in these datasets are then imputed, using a stochastic algorithm that estimates values based on information contained within the observed values and creates different values in each dataset. The additional variance caused by differences in the imputed values between the various copies reflects the uncertainty of the imputation. The relative increase of variance due to substitution of missing data can be calculated for any given data [5]. Statistics are performed separately for these datasets and coefficients are combined at the conclusion of the analysis [5]. Finally, the degrees of freedom are adjusted. MI leads to results without bias for many missing at random [MAR: [1] conditions that introduce bias in CC, but there are also situations in which MI produces bias and CC does not *e.g.*[6,7].

In most cases, it has been demonstrated that MI is superior to CC and various other ways of dealing with missing data *e.g.*[8]. This has been proven in large samples *e.g.*[9,10] or when data in the response variables is missing, for example when there are dropouts in repeated measurement designs *e.g.*[11]. In the latter case, variables from different points in time are often highly correlated, enabling MI algorithms to generate good results. In such complex responses consisting of many correlated variables, substitution of missing data generally improves parameter estimation. When missing data occurs in a single response (typically analysed within the general linear model), any way of substituting a response will introduce noise rather than being helpful if auxiliary variables are not used [12].

An additional advantage of MI over CC is the possibility of including information from auxiliary variables into the imputation model. Auxiliary variables are variables within the original data that are not included in the analysis, but are correlated to the variables of interest or help to keep the missing process random [MAR: [1]. Little [6] has calculated the amount of decrease in variance of a regression coefficient Y on X_1_ when a covariate *X*_2_ is added that has no missing data. White and Carlin [7] have extended this proof to more than one covariate. In practice however, it is likely that auxiliary variables themselves will have missing data. Collins *et al.*[13] performed a simulation study in which they tested the influence of auxiliary variables with missing data in a regression model. Particularly good results were obtained by including auxiliary variables when (a) the missing data were in the response, (b) the auxiliary variable changed the process leading to missing data from “missing not at random” (MNAR) to MAR and when (c) the correlation of the auxiliary variable to the response was very high, *i.e.* r=.9. In their study, the information gained due to auxiliary variables was generally larger than the noise that was introduced by irrelevant information. For this reason, they recommended using inclusive rather than restrictive strategies.

This is in line with recommendations by most experts: the imputation model should include all variables of the analysis, plus those highly correlated with responses or explanatory variables, and finally those variables that explain the mechanism leading to missing data *e.g.*[2,14]. Enders [15], p. 127ff] demonstrated that when an auxiliary variable which mediates between an outcome Y and an explanatory variable X is not included in the substitution model, some bias in the estimates occurs and the power of the analyses decreases.

Another simulation study explored the role of auxiliary variables in confirmatory factor analysis and also concluded that the inclusion of auxiliary variables is beneficial [16]. However, from a practical point of view, inclusion of too many variables leads to a considerable increase in computation time or even causes programs to fail [17]. The latter happens with continuous linear variables in most programs when the number of variables approaches the number of cases. In the study by Collins *et al.*[13], the ratio of subjects to included variables never fell below 10 to 1 – although this could easily occur in small sample research. For example, if a researcher had 100 subjects in a questionnaire survey comprising 10 scales based on six items each plus some demographics, there would be more than 60 variables on the item basis and about sixteen variables on the scale basis. Repeated measurement designs may have even more variables *e.g.*[18]. However, these face different problems that will not be addressed here.

The aim of the present paper is to simulate various realistic situations that might occur in a medical survey. Within this frame, we have explored the effect of using auxiliary variables in the substitution model for missing data in explanatory variables of a linear regression model. A model was chosen with a response Y with two correlated continuous predictors X_1_ and *X*_2_, both showing strong associations to Y total R^2^ = .40: [19]. Varying proportions of missing data were introduced into this model and analyses using CC and MI were applied, the latter with varying numbers of auxiliary variables. To illustrate, an example is provided where data is missing from the items of a questionnaire scale.

## Method

### Simulation design

The analysis parameter was the non-standardized b_1_-coefficient from the following formula:

$$Y=\alpha +b_{1}X_{1}+b_{2}X_{2}+e\text{,}$$

Where *X*_1_, *X*_2_ ∼ *N*(0, 1); *b*_1_, *b*_2_ = 1; *r*_*x*1*x*2_=.40, *e* ∼ *N*(0, *σ*^2^).

Results for the b_2_-coefficient were virtually identical and are not displayed. The simulation design was adopted from Allison [20] with some modifications, it was also utilized by Horton and Lipsitz [21]. Basically, we added the auxiliary variables, introduced additional random error into the MAR conditions and put missing values into all variables except the response, because we wanted to create typical conditions for small and moderate sample medical research. For the model described above, simulations with the following variations were conducted: (i) data sets with sample sizes of n = {50, 100, 200}, (ii) analyses utilizing CC or MI including a = {0,10,20,40,80} auxiliary variables Z_a_ which (iii) all have low (r = 0.1) or moderate (r = 0.5) correlations to Y, X_1_, *X*_2_ and all other Z_a+1_. In the simulations, (iv) 20% and 50% of the observations in the X_1_, *X*_2,_ and Z_a_ variables were deleted by (v) one of three mechanisms.

#### MCAR

Observations in X_1_, *X*_2_ and Z_a_ were deleted completely at random.

#### MAR(Y)

No full MAR mechanism was applied here, because in real data, finding a variable that completely explains the process of missingness is unlikely. Thus, the observations to be deleted in X_1_, *X*_2_ and Z_a_ were chosen depending on the unweighted sum of a random standard normal distribution and the standard normal distribution of Y, with the highest sums introducing missing data in X_1_, *X*_2_ and Z_a_. In terms of Allison (2000), this mechanism consists of “50% MCAR and 50% MAR(dependent on Y)”.

#### MAR(X)

A procedure similar to the one utilized in MAR(Y), but the observations of X_1_ and Z_a_ were deleted depending on the sum of *X*_2_ and a value from a random normal distribution; the observations of *X*_2_ depended on X_1_ and a value from a random normal distribution. Since X_1_ and *X*_2_ have a standard normal distribution as well, this results in “50% MCAR and 50% MAR(dependent on X)” as defined by Allison (2000). Again, the highest values introduced the missing data.

The generating process for MAR(Y) was:

After z-transformation of Y, let all X_1_, *X*_2_, Y and c be X_1_, *X*_2_, Y, c ~ N(0,1)

Summing: *d* = *Y* + *c*

Ranking: d_j_ < d_k_ for each i, j from N

Deleting: X_1_, *X*_2_, Z_a_ is missing if d ≥ h: for h = 4%, 8%…64%

Similar for MAR(X):

Let all X_1_, *X*_2_, Y, Z_a_, c ~ N(0,1)

Summing: *d*_1_ = *X*_2_ + *c*, *d*_2_ = *X*_1_ + *c*

Ranking: d_j_ < d_k_ for each i, j from N

Deleting: X_1,_ Z_a_ if d_1_ ≥ h: for h = 4%, 8%…64%

*X*_2_ if d_2_ ≥ h: for h = 4%, 8%…64%

No MNAR condition was created, and no observations of Y were deleted in the present study. The deletion of observation was carried out separately for each variable. This means that there were cases in the analyses that had no observed values for X_1_ and *X*_2_ as well as for Z_a_, only multiply imputed values. At first sight, one may question whether this makes sense. However, in a complex analysis where various regression analyses are involved (*e.g.* searching for a mediator), this could be necessary. Hence, our simulations encompassed this possibility.

Using multiple imputations by chained equations, m = 20 imputed data sets were created [1]. There was a recommendation by Little and Rubin that creating three to five data sets is usually enough. In more recent articles, probably due to increasing computational power, this recommendation has changed, suggesting the use of a greater number of imputed datasets in real data analyses, *i.e.* 20 – 100 [17,22,23]. Values were imputed *via* chained equations followed by predictive mean matching (PMM) using the program “MICE” [24,25]. This combination was chosen because it yielded the best results in previous simulation studies [26,27].

The combination of (i) three sample sizes, (ii) ten ways of handling missing data including the two levels of correlations (r=.1 or r=.5) in the auxiliary variables, (iv) two percentages of missing observations and (v) three mechanisms for deleting data resulted in 180 different simulations. The simulations were conducted with q=1000 replications each. Each simulation was started with a random seed and created different seeds for each replication for use with the random number generator. For each replication, a new complete dataset was generated, and then the intended percentage of data was deleted. Depending on the simulated condition, this was either directly followed by fitting the linear regression model using the CC method, or by first creating multiple imputations and then following with regression analyses. The latter were conducted separately on each data set and pooled applying Rubin’s Rules. For the b_1_-coefficient, the raw bias (mean of estimated – true coefficient), its standard error (SE), the standardized bias (raw bias/SE) as well as the root mean square error (RMSE) over the q=1000 replications (*i.e.*$\sqrt{\frac{{\left(bi-b\right)}^{2}}{q}}$ as an estimate for precision (b_i_ is the estimated b-coefficient for replication i, b the coefficient in the complete data, *i.e.* 1, and q the number of replications) are displayed in Tables 1, 2, 3. The presentation of results was oriented on Lee and Carlin’s [28], but the parameter for precision, coverage, was replaced by the RMSE.

**Table 1:** Results of the simulations on linear regressions, MAR(Y)

**Percent missing**		**Auxiliary variable correlation**	**n = 50**				**n = 100**				**n = 200**			
			**Bias**	**SE**	**Std. Bias**	**RMSE**	**Bias**	**SE**	**Std. Bias**	**RMSE**	**Bias**	**SE**	**Std. Bias**	**RMSE**
20	CC	-	−0.13	0.33	−0.43	0.20	−0.13	0.22	−0.60	0.17	−0.13	0.15	−0.85	0.15
	MI+0	-	−0.05	0.32	−0.11	0.16	−0.03	0.22	−0.10	0.11	−0.02	0.15	−0.09	0.08
	MI+10	0.1	−0.08	0.33	−0.21	0.17	−0.04	0.22	−0.17	0.10	−0.02	0.15	−0.10	0.07
	MI+20	0.1	−0.16	0.35	−0.43	0.21	−0.06	0.23	−0.26	0.12	−0.03	0.15	−0.16	0.07
	MI+40	0.1	−0.28	0.35	−0.77	0.32	−0.17	0.24	−0.66	0.19	−0.05	0.16	−0.32	0.08
	MI+80	0.1	−0.25	0.35	−0.70	0.31	−0.30	0.27	−1.13	0.33	−0.16	0.17	−0.94	0.17
	MI+10	0.5	−0.06	0.45	−0.10	0.23	−0.03	0.30	−0.06	0.15	0	0.21	0	0.11
	MI+20	0.5	−0.13	0.46	−0.25	0.26	−0.05	0.30	−0.14	0.17	−0.02	0.21	−0.07	0.10
	MI+40	0.5	−0.20	0.44	−0.46	0.33	−0.12	0.33	−0.37	0.19	−0.04	0.21	−0.16	0.11
	MI+80	0.5	−0.21	0.44	−0.45	0.34	−0.43	0.33	−1.37	0.43	−0.12	0.23	−0.53	0.16
50	CC	-	−0.14	0.57	−0.32	0.44	−0.25	0.35	−0.72	0.34	−0.23	0.25	−0.96	0.27
	MI+0	-	−0.13	0.44	−0.20	0.33	−0.08	0.29	−0.18	0.23	−0.06	0.21	−0.20	0.17
	MI+10	0.1	−0.27	0.47	−0.52	0.38	−0.13	0.30	−0.36	0.22	−0.07	0.20	−0.29	0.15
	MI+20	0.1	−0.59	0.48	−1.30	0.60	−0.23	0.33	−0.68	0.28	−0.10	0.21	−0.43	0.15
	MI+40	0.1	−0.52	0.42	−1.28	0.59	−0.57	0.33	−1.82	0.57	−0.21	0.23	−0.94	0.23
	MI+80	0.1	−0.50	0.42	−1.21	0.58	−0.54	0.29	−1.95	0.59	−0.56	0.23	−2.54	0.56
	MI+10	0.5	−0.23	0.63	−0.33	0.42	−0.09	0.41	−0.15	0.32	−0.03	0.28	−0.06	0.17
	MI+20	0.5	−0.50	0.59	−0.90	0.52	−0.18	0.44	−0.39	0.31	−0.06	0.28	−0.16	0.21
	MI+40	0.5	−0.45	0.50	−0.94	0.61	−0.51	0.41	−1.31	0.51	−0.18	0.31	−0.57	0.25
	MI+80	0.5	−0.43	0.51	−0.89	0.61	−0.43	0.35	−1.28	0.57	−0.51	0.29	−1.85	0.51

**Table 2:** Results of the simulations on linear regressions, MAR(X)

**Percent missing**		**Auxiliary variable correlation**	**n = 50**				**n = 100**				**n = 200**			
			**Bias**	**SE**	**Std. Bias**	**RMSE**	**Bias**	**SE**	**Std. Bias**	**RMSE**	**Bias**	**SE**	**Std. Bias**	**RMSE**
20	CC	-	−0.14	0.33	−0.43	0.20	−0.13	0.22	−0.60	0.17	−0.13	0.15	−0.85	0.15
	MI+0	-	−0.04	0.31	−0.12	0.15	−0.05	0.22	−0.22	0.10	−0.05	0.15	−0.32	0.08
	MI+10	0.1	−0.08	0.32	−0.24	0.16	−0.07	0.22	−0.30	0.12	−0.06	0.15	−0.36	0.08
	MI+20	0.1	−0.17	0.33	−0.51	0.21	−0.10	0.22	−0.46	0.13	−0.07	0.15	−0.44	0.09
	MI+40	0.1	−0.16	0.33	−0.48	0.21	−0.19	0.23	−0.85	0.21	−0.11	0.15	−0.68	0.11
	MI+80	0.1	−0.15	0.33	−0.45	0.21	−0.22	0.24	−0.93	0.23	−0.21	0.16	−1.34	0.21
	MI+10	0.5	−0.02	0.42	−0.02	0.19	−0.01	0.29	−0.03	0.13	−0.02	0.20	−0.08	0.09
	MI+20	0.5	−0.08	0.43	−0.15	0.20	−0.03	0.29	−0.10	0.13	−0.02	0.20	−0.10	0.09
	MI+40	0.5	−0.08	0.42	−0.19	0.23	−0.08	0.29	−0.26	0.15	−0.04	0.20	−0.17	0.10
	MI+80	0.5	−0.09	0.41	−0.21	0.23	−0.31	0.30	−1.11	0.32	−0.09	0.21	−0.43	0.12
50	CC	-	−0.14	0.57	−0.32	0.44	−0.25	0.35	−0.72	0.34	−0.23	0.25	−0.96	0.27
	MI+0	-	−0.20	0.46	−0.37	0.35	−0.21	0.31	−0.63	0.27	−0.18	0.21	−0.82	0.21
	MI+10	0.1	−0.28	0.47	−0.59	0.35	−0.23	0.31	−0.74	0.28	−0.20	0.21	−0.95	0.22
	MI+20	0.1	−0.53	0.44	−1.30	0.54	−0.27	0.30	−0.89	0.30	−0.23	0.21	−1.12	0.25
	MI+40	0.1	−0.42	0.42	−1.07	0.47	−0.52	0.30	−1.82	0.52	−0.28	0.21	−1.36	0.29
	MI+80	0.1	−0.44	0.42	−1.11	0.47	−0.44	0.28	−1.73	0.46	−0.53	0.20	−2.77	0.53
	MI+10	0.5	−0.12	0.6	−0.17	0.35	−0.10	0.41	−0.20	0.28	−0.07	0.28	−0.21	0.19
	MI+20	0.5	−0.41	0.55	−0.79	0.44	−0.11	0.40	−0.24	0.25	−0.09	0.28	−0.30	0.19
	MI+40	0.5	−0.30	0.51	−0.65	0.44	−0.38	0.38	−1.07	0.39	−0.14	0.28	−0.49	0.20
	MI+80	0.5	−0.28	0.51	−0.60	0.42	−0.21	0.36	−0.76	0.44	−0.38	0.25	−1.58	0.38

**Table 3:** Results of the simulations on linear regressions, MCAR

**Percent missing**		**Auxiliary variable correlation**	**n = 50**				**n = 100**				**n = 200**			
			**Bias**	**SE**	**Std. Bias**	**RMSE**	**Bias**	**SE**	**Std. Bias**	**RMSE**	**Bias**	**SE**	**Std. Bias**	**RMSE**
20	CC	-	0.02	0.34	0.06	0.19	0.01	0.24	−0.05	0.12	−0.01	0.16	−0.05	0.08
	MI+0	-	0	0.30	0.01	0.12	0	0.20	0.01	0.09	0	0.14	−0.01	0.06
	MI+10	0.1	−0.06	0.31	−0.17	0.14	−0.04	0.21	−0.16	0.09	−0.01	0.14	−0.06	0.06
	MI+20	0.1	−0.16	0.32	−0.49	0.19	−0.06	0.21	−0.25	0.11	−0.02	0.14	−0.14	0.06
	MI+40	0.1	−0.13	0.33	−0.41	0.18	−0.17	0.22	−0.75	0.18	−0.06	0.15	−0.42	0.08
	MI+80	0.1	−0.15	0.32	−0.46	0.19	−0.19	0.25	−0.77	0.20	−0.17	0.15	−1.09	0.17
	MI+10	0.5	−0.03	0.40	−0.06	0.18	0	0.27	0.01	0.11	0	0.19	−0.01	0.08
	MI+20	0.5	−0.06	0.40	−0.13	0.19	−0.03	0.28	−0.09	0.12	−0.02	0.19	−0.08	0.08
	MI+40	0.5	−0.09	0.41	−0.23	0.21	−0.07	0.28	−0.24	0.13	−0.03	0.19	−0.15	0.09
	MI+80	0.5	−0.06	0.40	−0.14	0.20	−0.30	0.27	−1.13	0.30	−0.08	0.19	−0.42	0.11
50	CC	-	0.12	0.62	0.18	0.43	−0.02	0.38	−0.08	0.28	0.04	0.27	0.15	0.19
	MI+0	-	−0.04	0.40	−0.04	0.28	−0.01	0.26	0.04	0.18	−0.01	0.18	−0.01	0.12
	MI+10	0.1	−0.20	0.43	−0.44	0.31	−0.10	0.28	−0.34	0.20	−0.04	0.18	−0.19	0.13
	MI+20	0.1	−0.49	0.42	−1.25	0.50	−0.18	0.29	−0.64	0.22	−0.10	0.19	−0.52	0.14
	MI+40	0.1	−0.35	0.41	−0.94	0.40	−0.49	0.29	−1.77	0.49	−0.19	0.19	−1.02	0.20
	MI+80	0.1	−0.33	0.40	−0.89	0.38	−0.35	0.28	−1.38	0.38	−0.48	0.20	−2.55	0.48
	MI+10	0.5	−0.06	0.53	−0.09	0.31	0	0.35	0.06	0.23	−0.01	0.24	0	0.16
	MI+20	0.5	−0.34	0.51	−0.72	0.37	−0.03	0.36	−0.07	0.21	−0.03	0.24	−0.10	0.15
	MI+40	0.5	−0.20	0.47	−0.48	0.35	−0.31	0.35	−0.91	0.32	−0.06	0.24	−0.26	0.15
	MI+80	0.5	−0.22	0.48	−0.51	0.36	−0.19	0.33	−0.68	0.31	−0.30	0.24	−1.31	0.30

**Figure 1:**
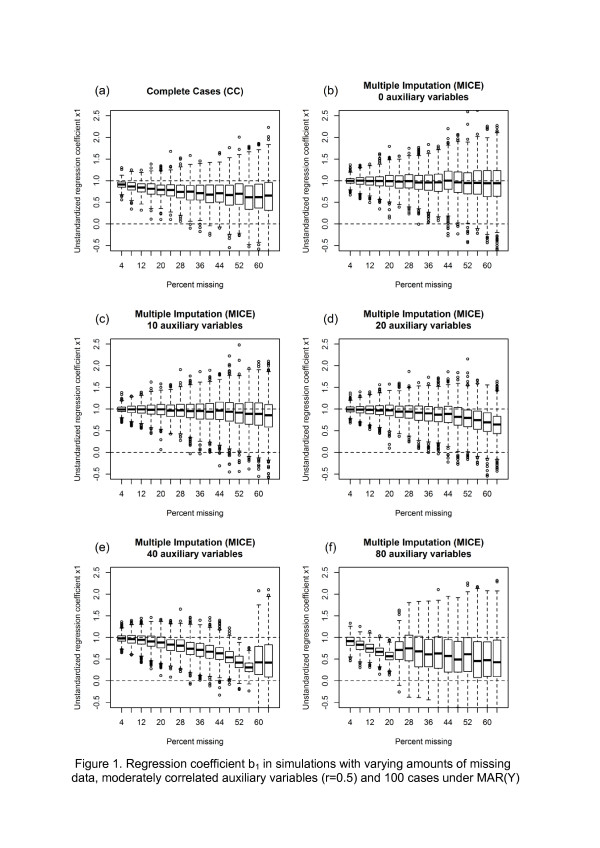
**Regression coefficient b**_**1 **_**in simulations with varying amounts of missing data, moderately correlated auxiliary variables (r=0.5) and 100 cases under MAR(Y).**

For illustrative purposes, a first set of simulations was performed on a dataset with a sample size of n=100, again utilising CC and MI with 0 to 80 auxiliary variables and moderate correlations under the MAR(Y) mechanism as described above. In these simulations, data was deleted for exactly h = 4, 8, 12, …, 64% of the observations for X_1_, *X*_2,_ and all Z. Again, a total of 1000 replications for each rate of missing data were performed - everything else followed the design presented above, but the b_1_-coefficient is shown in Figure 1 using box plots.

### Data generation and selection of program parameters

Data was generated in three steps. First, X_1_ and *X*_2_ were created in random order, drawn from a normal distribution. Second, Y was generated by drawing from a normal distribution, setting b_1_ and b_2_-coefficients to 1, then obtaining the necessary errors and adding those to Y so that an R^2^ of .40 could be created. Third, the auxiliary variables Z were created in random order. Using the Choleski factorization, the variables were transformed to show the desired correlation matrix (all coefficients r = .1 or .5) was generated.

All simulations were performed using R [29]. The simulation data were generated utilizing R's methods for generating random, normally distributed variables, applying the Choleski factorization and solving systems of linear equations [rnorm, chol, solve: [30], all taken from the base and stats packages. R's default random number generator "Mersenne-Twister" was used. The seeds used for each simulation were true random numbers provided by random.org *via* the package “random” [randomNumbers: [31]. Multiple imputation was also performed in R using the package MICE V2.0: [25]. The script used to create the simulation data, induce missing data and perform multiple imputations and regression analyses can be obtained from the authors.

## Results

Figure 1 displays the non-standardized regression coefficient b_1_ under MAR(Y) and n=100. It illustrates the curve that occurs with increasing amounts of missing data with various numbers of auxiliary variables. The horizontal axis of the graph stands for the percentage of missing data; the vertical axis displays the distributions of the coefficient b_1_ over the 1000 replications for each proportion of missing data. The box-and-whisker plots display the box as the usual 25%, 50%, and 75%-quantiles of b_1_ with the whisker having a maximum of 1.5 times the length of the box. Outlier values of b_1_ are displayed as circles. The line at value 1 represents the true regression coefficient when there are no missing data. All auxiliary variables in Figure 1 have moderate correlations of r=.5 to X_1_, *X*_2_, Y and all Z.

Figure 1a displays the results of the CC analysis. A downward bias is clearly visible with increasing rates of missing values, as well as a decrease in precision. Figure 1b shows the same simulation after multiple imputations without any auxiliary variables, *i.e.* only Y, X_1_ and *X*_2_ are in the imputation model (MI+0). Compared to CC, there was almost no bias and precision was minimally higher.

Figure 1c displays the situation when ten auxiliary variables were added (MI+10). Here, a slight downward trend of the coefficient could be observed when the missing rate exceeded 40%. Precision was somewhat better than in MI+0. Figure 1d displays the same simulation with 20 auxiliary variables (MI+20). Up to a missing data rate of 20%, bias was small and precision did not improve compared to MI+10. However, with higher rates of missing data, bias increased. Figures 1e and f display the results after inclusion of 40 and 80 auxiliary variables, respectively. They clearly indicate that the inclusion of too many variables did not improve the imputation process under these conditions; rather, it was disadvantageous. The inclusion of 40 variables may be acceptable when very few data (<10%) are missing, but it was clearly worse than using less auxiliary variables. The inclusion of 80 auxiliary variables did not make sense in this simulation; when 8% of the data was missing, there was already an extreme bias so that an analysis performed on that data set would be seriously impaired by the multiple imputation.

Table 1 displays the results of the main simulations under MAR(Y). The first column under each sample size displays the raw bias of b_1_ over q = 1000 replications. It ranges between zero and -.59 in these simulations, no upward bias was observed, here. Raw bias was not positively affected by the inclusion of auxiliary variables in these simulations except for one case: 50% missing data, n=200 and 10 auxiliary variables, where it is reduced from -.06 for MI+0 to -.03 for MI+10. The second column displays its standard error (SE) which decreases with the sample size and increases with the number of auxiliary variables. The third column displays the standardized bias, *i.e.* the ratio of the former two. With few exceptions, the standardized bias increases with the number of auxiliary variables, indicating that including auxiliary variables is not beneficial in these simulations. The exceptions are: ten auxiliary variables with a correlation of r = .5 for n = 50, 100, and 200 with 20% missing data, ten auxiliary variables for n = 100 and 200 with 50% missing data, and 20 auxiliary variables for n = 200 in 20% as well as for 50% missing data. The fourth column displays the root mean square error (RMSE), which shows lowest values in most cases for the MI+0 condition. In sum, MI+0 performs much better than CC, but the inclusion of auxiliary variables is not a great advantage under the MAR(Y) condition realized here.

Table 2 displays a similar simulation, but an MAR(X) condition was realized. The basic patterns are similar to Table 1. MI generally performed better than CC, bias and SE tended to increase with the number of auxiliary variables, the same holds true for the RMSE. However, there is one difference to MAR(Y): the inclusion of ten auxiliary variables with r =.5 was helpful in all sample sizes, 20 variables with n≥100, and 40 variables with n = 200. Including more variables caused a downward bias of coefficients and precision decreased as was observed under MAR(Y). For auxiliary variables with low correlations, no such effect was observed.

Table 3 displays a simulation where an MCAR condition was realized. The basic patterns are different from Tables 1 and 2. Naturally, there was no bias under CC, so MI could not perform better than CC bias. Also, no benefit from including auxiliary variables could be observed regarding precision. However, including few auxiliary variables did not cause damage: ten auxiliary variables did not introduce bias for n ≥ 100 for 20% missing data, and for n = 200 also for 50% missing data – precision was only slightly worse then. Similar to the two MAR conditions, including too many auxiliary variables caused a downward bias of coefficients and a loss of precision.

A subset of simulations was replicated using different algorithms and programs. Some of the simulations were repeated using two different joint modelling algorithms, Norm [23,32] and Amelia II [33]. Results were basically the same as those presented here, except that in small samples, the curves were less smooth when compared with the MICE algorithm. A similar result was reported by Taylor and Zhou [34]. Both programs, Amelia and Norm, broke down when the number of auxiliary variables and the missing rate was high and the sample size low. For example, in samples of n = 100 and 20% missing data, not more than about 40 auxiliary variables could be included. Three other programs based on the MICE algorithm, STATA’s V12 [23], ICE [35] and STATA’s “MI” [23] utilizing “chained (pmm)” and SPSS [36], basically led to the same results as R’s MICE. However, they also broke down when the number of auxiliary variables (minus one) reached the number of cases with data. This is no disadvantage over MICE, given the results of the present simulation study. As this paper outlines, it makes no sense to reach a point where the number of variables is equal to the number of cases.

To explore if the present simulation results can be generalized to larger samples, we performed a simulation as defined above, having 50% missing data, MAR(Y), but 350 auxiliary variables, all r = .5, n = 1000. To reduce the computational time, only m = 5 imputed datasets were created and only 200 replications performed. Results show a strong downward bias under MI+350 (bias = −.53, SE = .26, std. bias = −2.04, RMSE = .54), even somewhat worse than under CC (bias = −.37, SE = .25, std. bias = −1.48, RMSE = .39), compared to MI+0 (bias = −.01, SE = .34, std. bias = −.03, RMSE = .17).

Marshall *et al.*[27] compared various algorithms (among them MICE and various joint modelling algorithms) in a simulation with eight predictors in a logistic regression with n=1000 subjects and did not find large differences. We would draw the conclusion that (1) the present effects of auxiliary variables were not the result of a specific program and that (2) in small samples, the MICE algorithm seems to perform better. However, in large samples, one of the – usually much faster – joint modelling algorithms would probably be preferable.

### Example

In addition to the simulations, an example utilizing real data is provided. The data for this example is taken from an online survey, which was conducted in 2008 [37] in order to cross-validate a questionnaire, the SCL-27-plus. It is used to screen for symptoms of depression, agoraphobia, social anxiety, pain and vegetative symptoms. Questionnaires on quality of life [38] and parent–child relationships were also included [39]. For the present example, a score based on 8 items from the physical functioning scale of the quality of life (QOL) instrument was chosen as the response, and the pain score (six items) of the SCL-27-plus as a predictor (PAIN). In total, 48 additional items were used as auxiliary variables. Out of 500 cases, 100 were randomly chosen for this example. None of the cases had any missing data in QOL, PAIN or any auxiliary variable.

For the example, we conducted a simple regression analysis. The unstandardized b-coefficient for PAIN on QOL was −1.97 with a standard error of 0.71 and a t-value of −2.76 in the 100 cases with complete data. As in the simulations, successively increasing numbers of observations (5%, 10% … 50% from all 40 items except the 8 for QOL were deleted using a 50% MAR(Y) and 50% MCAR mechanism – the higher the QOL, the higher the probability the items were missing. After each step, two multiple imputations were performed and the impact on the following regression analysis examined. The first multiple imputation only used QOL and the six items of the score for PAIN for its imputation model (MI+0), the second used all other available variables as auxiliary variables for its imputation model (MI+48). The bivariate correlations between the variables were .23 on average, with the lowest being close to 0, and the highest .82. Program settings were identical compared to the simulated data.

When 5% of the observations were deleted, whether auxiliary variables were included or not made no visible difference. Both showed negligible bias with standard biases of −0.01 and 0.03, as compared to the value over the complete data. When the proportion of missing data rose, using no auxiliary variables for the imputation model did not increase bias. When 50% of the observations were deleted and substituted, the relative bias was still less than 5% and the standard bias was only slightly increased to −0.08. Utilizing the 48 auxiliary variables introduced considerable bias with higher proportions of missing data. With 50% of the data missing, the standardized bias reached 0.99 and the mean b-coefficient was clearly biased towards zero.

**Figure 2:**
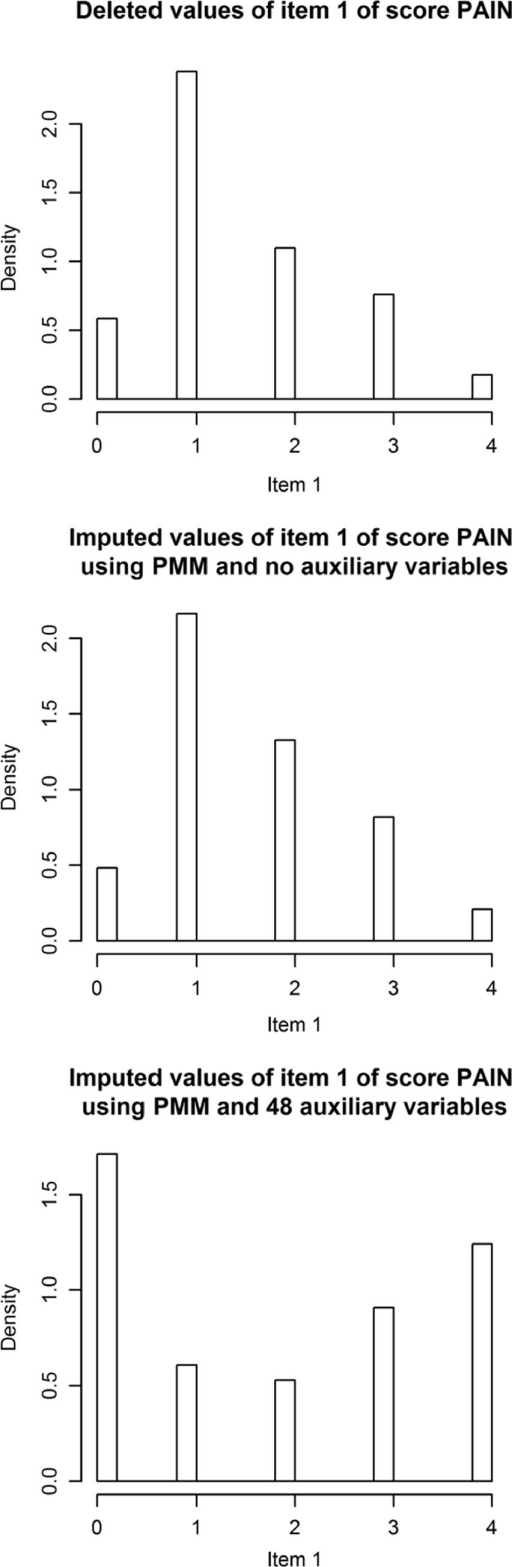
Comparison of deleted values and imputed values, using PMM and no or 48 auxiliary variables in an exemplary dataset.

Figure 2 examined what happened when too many auxiliary variables were in the imputation model. The first item of the PAIN score is used for demonstration. The top figure shows the distribution of answers from 50 remaining cases when the other 50 were deleted. Because the missing mechanism is partly MAR, the first distribution is not an approximation for the distribution of the complete data, but skewed. The second graph shows the distribution of the values that were imputed using no auxiliary variables. Virtually no difference from the first graph can be observed. The third graph shows the distribution of imputed values using all 48 auxiliary variables. It clearly deviates from the original data, with a much broader distribution.

## Discussion

The results of the present simulation study can be summarized very briefly: In MI, inclusion of some auxiliary variables may help, too many can be harmful. Under MCAR, the inclusion of auxiliary variables was worthless and under MAR(Y), advantages were limited. The best results here were observed under the MAR(X) condition, where a reduction of bias plus an increase of precision could be reached by including a few auxiliary variables. Further, few auxiliary variables did not cause harm within the simulations realized here. The reason why too many variables introduce bias is probably that regression models become unstable when the ratio of cases to variables gets low due to over-parameterization of the imputation model. Standard textbooks usually recommend having at least ten cases per variable in regression models. From the present simulations, we derived a preliminary rule of thumb that is somewhat below the lower limit of such recommendations – not to include a larger number of variables than 1/3 of the cases with complete data for the variable to be imputed. An optimal imputation model would have fewer variables, but including up to 1/3 would not do much harm (to continuous variables, see below). This relativizes recommendations from other sources, who mostly recommend including more rather than fewer auxiliary variables. These other recommendations should by no means be discredited by our simulation, they are correct - we merely want to show that the number of auxiliary variables that are included has an upper limit. We assume that the authors of these recommendations consider it obvious that over-parameterization needs to be avoided and therefore did not focus on it.

One of the main sources cited in these recommendations is the simulation performed by Collins *et al.*[13]. We found five main differences between the Collins *et al.* simulations and ours.

(1) There were no missing values in our response; Collins *et al.*[13] observed the best effects of including auxiliary variables when the missing data were in the response. Hence, we included missing data in our response but still could not attribute a positive effect to auxiliary variables.

(2) Our auxiliary variables had correlations of r=.1 and r=.5, Collins *et al.*[13] used one variable with a correlation of r=.4 or r=.9. We were able to replicate the results from Collins *et al.* when including one variable with a correlation of r=.9. However, we did not want to use such high correlations because they are not likely to be observed in clinical research. When we performed simulations with correlations of r=.7 and higher, a stronger positive effect of including a few auxiliary variables could be observed. Particularly with relatively small amounts of missing data, we observed higher precision, with the drawback that larger amounts of missing data introduced bias. This data is not shown here because it is not realistic to assume such high correlations in real data. This result is congruent with a simulation performed by Enders and Peugh [40] who included six auxiliary variables into a factor analysis on samples with missing data rates of up to 25%. In this study, factor loadings were .60 - .70, and the correlations of the auxiliary variables were all r=.3. No substantial benefit from the auxiliary variables was observed.

(3) Collins *et al.* had no missing data in the auxiliary variables themselves. Hence, we also performed a simulation where only the X’s had missing data. Results were very similar to the ones obtained by correlations of r=.7, *i.e.* a slightly positive effect of including a few auxiliary variables was observed. With relatively small amounts of missing data, a higher precision was achieved, but again, with larger amounts of missing data, some bias occurred.

(4) A further difference between our study and the simulations performed by Collins *et al.*[13] was that in the latter, the auxiliary variable Z was associated with the likelihood for Y to be missing, *i.e.* an MNAR condition was introduced when the auxiliary variable was omitted. Hence, we performed a similar simulation, but in ours the value of Z was associated with the likelihood of X_1_ and *X*_2_ being missing. As a result, no positive effect of including this variable Z could be observed.

(5) Collins *et al.*[13] did not focus solely on a regression coefficient as we did here, but also examined means, standard deviations and correlations. They observed stronger effects of the auxiliary variables on means and standard deviations than on regression coefficients.

From this, we can conclude that in estimating a linear regression coefficient, (1) it doesn’t matter whether missing values are in the explanatory variables only or also in the response, (2) inclusion of auxiliary variables is most helpful when the correlations to the X’s and Y are high (*i.e.* r ≥ |.5|), (3) auxiliary variables without missing data perform a little better than those that have missing data themselves, (4) a variable that explains the mechanism leading to missing data in the predictors need not necessarily be included in the imputation model and (5) regression coefficients are less sensitive to bias than means and standard deviations. Conclusion (4) in particular came as a surprise to us, because it partially contradicts the intuitively appealing and well-accepted recommendation to keep the data MAR [14]. Further research is necessary to explore under which conditions it is beneficial to include a variable to keep the data MAR or whether including such a variable is disadvantageous. Conclusion (2) is similar to one provided by Enders [15], who has suggested that correlations greater than ± .40 are generally helpful.

A noteworthy effect was the drastic breakdown of the regression coefficient when too many variables were included, as displayed in Figures 1e and f. Therefore, we analyzed the distribution of the imputed values for Figure 1f when only 4% of the cases were missing (4 missing values × 20 imputed datasets × 1000 replications = 80,000 data points). It turned out that there was an extreme variation in the imputed values – the standard deviation was 8 under this condition, instead of 1 as it was in the “observed” values. We repeated this analysis in our real data example with a higher percentage of missing data and obtained the same result. An almost normal distribution of complete data changed to a U-shape by imputation with too many auxiliary variables.

In principle, this simulation study confirmed the observation that under realistic conditions, a small amount of missing data (*e.g.* <5%) usually does not lead to severe bias or relevant loss in precision regardless which method of dealing with missing data is applied [27], in longitudinal studies, even larger missing rates can be tolerable [41]. However, when missing rates become higher and too many auxiliary variables are included in the imputation model, the regression coefficient can becomes seriously biased downward and imprecise (Figure 1e and f). If multiple imputations are used at a ratio close to 1:1 of variables and cases, *i.e.* the point where chained equation programs often break down, even with small rates of missing data (*i.e.* 5-10%), coefficients can be seriously biased.

With the present simulations, we have tried to create conditions that are typical for data analyses in medicine and life sciences, *i.e.* small to moderate sample sizes, small to moderate correlations among the variables and small to moderate amounts of missing data. For small numbers of auxiliary variables, we saw an almost linear increase in bias and a decrease in precision with growing rates of missing data (Figure 1). However, as a rule of thumb, we suggest restricting the number of auxiliary variables to not more than 1/3 of the cases with complete data, *i.e.* all cases minus those with missing data [5]. As an example, with 100 cases and 40% missing data, 60 cases have complete data. Hence, no more than 60/3 = 20 variables should be used in the imputation model. This holds true for continuous variables, and will not dramatically change when a few explanatory binary variables are in the model. Binary responses or datasets consisting mainly of binary or categorical variables with more than two categories will need a higher variables/cases ratio – a simulation study on this is currently being planned? Using fewer variables should not be problematic, while using more variables would cause the risk for a serious downward bias of the regression coefficients. This was tested for samples of n=50, 100, and 200 under MCAR and two MAR mechanisms, including auxiliary variables with low or moderate correlations, and missing rates of up to 64%. Within the limits studied here, sample size does not indicate a strong deviation from a “not more than 1/3” rule, higher correlation of auxiliary variables are better under MAR conditions, and MAR(X) profits more form auxiliary variables than MAR(Y).

The result can be plausibly extrapolated for larger samples – our simulation with n=1000 and 350 variables showed a downward bias, too. However, we did not systematically perform simulations with larger n’s, because a large n here results in large numbers of auxiliary variables, which leads to long computational times. How far the 1/3 rule can be extrapolated to rates higher than 64% of missing data was intentionally not tested. Situations may exist in which a substitution of very large amounts of missing data (*e.g.* 90%) makes sense. In our simulations, it did not. Marshall *et al.*[26,27] warned that using a multiple imputation that works well with less than 50% missing data introduces bias in a Cox regression when 75% of the data were missing. We do not recommend applying our rule to proportions of missing data larger than 64% without performing further simulations.

What to do when many variables are utilized in an analysis with a limited sample size? Or if a statistician wants to impute the missing data in a set to make it freely usable for public – in this case, he cannot know which statistical methods will be applied later. In such situations, including more variables than three times the number of cases with complete data may be desirable – even without considering any auxiliary variables. Then the choice of a program that allows a restriction of the number of predictors for each variable for which imputations are to be done is recommended – mostly this would be a MICE algorithm. This helps to avoid over-parameterization. Different possibilities in the various programs exist. Some (*e.g.* R’s mice, STATA) offer convenient ways to define which variables are used as predictors for imputations of other variables, others currently would make it difficult to do so (*e.g.* SPSS). If, further, the dataset consists of items belonging to different sub scales of a questionnaire for example, it would make sense to use this information and to impute each subscale separately. If such a structure is not present, one should try to maximize the squared multiple correlations “using as few auxiliary variables as possible” [15], p. 133. With STATA [23], such a selection needs to be done manually, R’s mice provides a tool that automatizes this task quickpred: [25,42]. The predictor matrix can be displayed to see the selected variables and modified if desired.

Simulation studies should always be read with care. (1) We have only studied linear regression coefficients here. Other statistical parameters may be influenced differently by auxiliary variables. For example, in simulations by Hoo [16], positive effects on bias through inclusion of auxiliary variables were seen in some standard errors in a confirmatory factor analysis, though the factor loadings themselves were not affected. (2) The present simulations are restricted to continuous data. Analysis of categorical data will introduce additional challenges. In that case, it is not only to be expected that the ratio variables/number of cases with complete data would be smaller, but the number of events per variable [43,44] will probably also become a parameter of interest. (3) In our simulations, all variables were distributed normally and relations were perfectly linear. Both are not likely to happen in real data. Deviations from normal distribution may have a negative effect on the MI process, which should be examined in further studies [45]. Problems of including quadratic or interaction effects were examined by Seaman *et al.*[46] and van Buuren [42]. (4) In large surveys where thousands of cases are collected, even large numbers of variables may become a problem. This was not examined in detail here due to the limitations of computational power. Creating Figure 1f, for example, required more than 200 hours of computing time on a 6 physical core PC optimized for these simulations, and estimate of 350 variables and 1000 cases about 60 hours. (5) We have displayed the distributions of the beta-coefficient only. This was done because research often focuses on the analysis of associations between variables. Simple point estimates of prevalence or means seem to benefit more from the inclusion of auxiliary variables. (6) Our simulations display downward bias. This is not necessarily always the case. Knol *et al.*[47] created four scenarios where down- and upward bias occurred under CC. Whether such upward bias, *i.e.* overestimation of an association, can also occur under a multiple imputation is unknown.

To summarize, we have learned from the present simulation that in a typical life science survey, the inclusion of auxiliary variables is often of little use; too many auxiliary variables may even be disadvantageous. Unless the correlations are high, we recommend keeping the number of variables in the imputation model as low as possible; even variables that explain the mechanism leading to missing data don’t necessarily need to be included. In our initial example, we used a researcher with 100 subjects that had ten scales based on six items each plus some demographics. This researcher was considering whether it would make more sense to substitute missing data on the item or on the scale level. Based on this example, our recommendation would be to impute in sub-models and to carefully select the variables, rather than using one imputation model for all data.

## Conclusion

Inclusion of too many auxiliary variables can seriously bias estimates in regression. We suggest a rule of thumb: that the number of cases with complete data should be at least three times the number of variables – otherwise, restricting the number of predictors becomes an option. This holds true for datasets containing mainly continuous and some binary variables. Performing MI in data sets consisting predominantly of categorical variables, maybe even with many categories, will be even more difficult in small and medium samples.

This work was funded in part by the Stiftung Rheinland-Pfalz für Innovation (Foundation Rhineland-Palatinate for Innovation: Az 959).

## Competing interests

The authors declare that they have no competing interests.

## Authors contributions

JH planned the paper and took a lead in writing it. MH carried out the simulations. RL planned the paper and corrected many details. All authors read and approved the final manuscript.

## Pre-publication history

The pre-publication history for this paper can be accessed here:

http://www.biomedcentral.com/1471-2288/12/184/prepub
